# Cold Induced Depot-Specific Browning in Ferret Aortic Perivascular Adipose Tissue

**DOI:** 10.3389/fphys.2019.01171

**Published:** 2019-09-18

**Authors:** Bàrbara Reynés, Evert M. van Schothorst, Jaap Keijer, Enzo Ceresi, Paula Oliver, Andreu Palou

**Affiliations:** ^1^Laboratory of Molecular Biology, Nutrition and Biotechnology, Universitat de les Illes Balears, Palma, Spain; ^2^CIBER de Fisiopatología de la Obesidad y Nutrición, Madrid, Spain; ^3^Institut d’Investigació Sanitària Illes Balears, Palma, Spain; ^4^Human and Animal Physiology, Wageningen University and Research, Wageningen, Netherlands

**Keywords:** adipose tissue, browning, cardiovascular disease, cold exposure, inflammation, thermogenesis

## Abstract

Brown adipose tissue is responsible for facultative thermogenesis to produce heat and increase energy expenditure in response to proper stimuli, e.g., cold. Acquisition of brown-like features (browning) in perivascular white adipose tissue (PVAT) may protect against obesity/cardiovascular disease. Most browning studies are performed in rodents, but translation to humans would benefit from a closer animal model. Therefore, we studied the browning response of ferret thoracic aortic PVAT (tPVAT) to cold. We performed global transcriptome analysis of tPVAT of 3-month-old ferrets acclimatized 1 week to 22 or 4°C, and compared the results with those of inguinal subcutaneous adipose tissue. Immunohistochemistry was used to visualize browning. Transcriptome data revealed a stronger cold exposure response of tPVAT, including increased expression of key brown/brite markers, compared to subcutaneous fat. This translated into a clear white-to-brown remodeling of tPVAT, with the appearance of multilocular highly UCP1-stained adipocytes. The pathway most affected by cold exposure in tPVAT was immune response, characterized by down-regulation of immune-related genes, with cardio protective implications. On the other hand, subcutaneous fat responded to cold by increasing energy metabolism based on increased expression of fatty acid oxidation and tricarboxylic acid cycle genes, concordant with lower inguinal adipose tissue weight in cold-exposed animals. Thus, ferret tPVAT responds to cold acclimation with a strong induction of browning and immunosuppression compared to subcutaneous fat. Our results present ferrets as an accessible translational animal model displaying functional responses relevant for obesity and cardiovascular disease prevention.

## Introduction

Adipose tissue has different biological functions, including adaptation to low ambient temperatures (Cinti, 2005). It is well known that cold acclimation, via beta-adrenergic stimulation, induces fatty acid catabolism in white adipose tissue (WAT), promoting both triacylglycerol lipolysis and fatty acid beta-oxidation, while decreasing fatty acid synthesis (Palou et al., 1998; Choe et al., 2016). In parallel, cold exposure activates brown adipose tissue (BAT), which uses circulating free fatty acids produced by catabolic lipid metabolism of WAT to produce heat representing non-shivering or facultative thermogenesis (Cannon and Nedergaard, 2004; Cinti, 2005). This process is mediated by the uncoupling protein 1 (UCP1) (Cannon and Nedergaard, 2004) present in the inner mitochondrial membrane of brown adipocytes, which acts as a proton conductor, dissipating the proton gradient generated by the respiratory chain as heat (Palou et al., 1998). Moreover, it is well known that cold exposure not only activates BAT but also results in a remarkable induction of UCP1-positive brown fat-like adipocytes in WAT, the so-called beige or brite cells (Ishibashi and Seale, 2010; Petrovic et al., 2010). This process, known as browning is widely characterized in rodents (Cousin et al., 1992; García-Ruiz et al., 2015) and could contribute to energy dissipation as heat. It is therefore not surprising that the detection in humans of cold inducible metabolically active brown fat has provoked an interest in the activation of brown and brite adipocytes as a potential anti-obesity therapeutic strategy (Nedergaard et al., 2007; Seale et al., 2011; Saito, 2013; Lee et al., 2014). BAT of adult humans is not well defined and is present in a dispersed manner in cervical, supraclavicular and paravertebral regions (Nedergaard et al., 2007). It has been proposed that, in humans, brown adipocytes could be, in fact, beige adipocytes, arising as result of WAT browning in response to proper stimuli (Wu et al., 2012).

Excess fat deposition around blood vessels, particularly the heart and coronary arteries is related to increased cardiovascular risk (Montani et al., 2004). In contrast, browning of this aortic perivascular adipose tissue (PVAT) has been related to an improved cardiovascular health (Aldiss et al., 2017; Xiong et al., 2017; Villarroya et al., 2018). Due to the difficulties of performing human studies, rodents are the most widely used species for thermogenic research (Bonet et al., 2013). Human thoracic PVAT (tPVAT) is comprised of brite adipocytes and, thus, has browning capacity (Chatterjee et al., 2009). However, in contrast to humans, rodent tPVAT presents more similarities with classical BAT, and its gene expression pattern and morphology is closer to that of BAT than to WAT (Fitzgibbons et al., 2011; Tran et al., 2018). Moreover, it has recently been reported that humans and mice present opposing browning gene expression patterns in visceral and subcutaneous adipose tissue depots (Zuriaga et al., 2017). In addition, contrary to what happens in humans, rodents possess a well-defined BAT, mainly located in the interscapular region. This fact could interfere when using this animal model to evaluate the relevance of browning itself, as most of the stimuli known to activate adipose tissue remodeling, usually also stimulate BAT thermogenesis (Bonet et al., 2013). Given the importance of understanding browning in humans, and because of the differences between human and rodent PVAT, there is an urgent need for another animal model that is closer to humans to perform BAT/browning studies.

Ferrets (*Mustela putorius furo*) is an animal model used in different research areas, e.g., immunology, due to their closer resemblance to humans than the most widely used rodents (Oh and Hurt, 2016; Stittelaar et al., 2016). Ferrets are closer to humans in terms of thermal environment, as the thermoneutral temperature for *Mustela putorius* has been established at 25°C (Korhonen et al., 1983), lower to that of rodents (28°C), and closer to that of non-naked humans (23°C) (Speakman and Keijer, 2012). This is of interest for thermogenic research, however, to our knowledge, our group is the only one studying browning phenomenon in this animal model. We have previously described that, unlike rodents, but similar to what is described in humans, adult ferrets do not present a well-defined BAT (Fuster et al., 2009; Sánchez et al., 2009). Noteworthy, these animals present dispersed multilocular adipocytes with modest levels of UCP1 in different adipose tissue depots, which increase by cold exposure and dietary stimuli, mainly in the retroperitoneal depot (Fuster et al., 2009; Sánchez et al., 2009). These data point to ferrets as an interesting alternative model to rodents to be used in studies of thermogenesis, as we have reported in a revision on white adipose tissue browning (Bonet et al., 2013). Moreover, we have previously shown in ferrets that cold exposure induces immunosuppression in tPVAT and in peripheral blood mononuclear cells (Reynés et al., 2017), which has been related with cardiovascular protection (Li et al., 2017). Given the relevance of PVAT for cardiovascular disease in humans and given our previous results, we here analyze, in the same set of animals, the browning response of this depot in the ferret to cold exposure, which is the main thermogenic stimulus. Moreover, using global gene expression analysis, we compared tPVAT to subcutaneous inguinal white adipose tissue (IAT) of ferrets acclimatized to 22 or 4°C during 1 week. We selected the IAT to compare because it is the one traditionally used for browning research in rodents (Wang and Yang, 2017), but according to our previous studies, in ferrets it could have a different cold-response adaptation, not related to browning (Fuster et al., 2009). Our study was complemented with morphological analysis to visualize functional adipose tissue responses.

## Materials and Methods

### Animal Procedure

Animal experiments followed in this study was reviewed and approved by the Bioethical Committee of the University of the Balearic Islands, and animal procedures followed the guidelines from the Directive 2010/63/EU of the European Parliament on the protection of animals used for scientific purposes. Three month-old male ferrets (*Mustela putorius furo* from Cunipic, Lleida, Spain) were distributed into two groups (*n* = 7): a control group, acclimatized to room temperature (22 ± 2°C), and a cold group, acclimatized to 4°C for 1 week. Ferrets are cold-exposed mammals in their natural environment, and while the optimum range recommended by the Council of Europe Convention for housing of ferrets is 15 to 22°C, they can live at ambient temperatures between 3 and 17°C (Fox, 1998). Thus, 1 week of cold exposure to 4°C is a strong cold stimulus, but not extreme for these animals. Thermoneutrality for polecats (*Mustela putorius*) is established at 25°C (Korhonen et al., 1983) and, therefore, we worked slightly below the thermoneutral temperature, which could be considered closer to the comfort zone. The animals were weighed before and after cold exposure. Due to logistic reasons, cold-exposed animals were housed individually to avoid huddling behavior to stay warm, and although they could see and smell each other, isolation could be considered as a potential confounding factor. All animals were exposed to a light/dark cycle of 12 h and had free access to water and diet (Gonzalo Zaragoza Manresa SL, Alicante, Spain). Ferrets were anesthetized using 10 mg/kg of Ketamine hydrochloride (Imalgène 1000, Merial Laboratorios SA, Lyon, France) and 80 mg/kg medetomidine (Domtor, Orion Pharma, Espoo, Finland), arterial blood was collected from the left ventricle and animals died by exsanguination. Afterward, thoracic perivascular, inguinal, interscapular and retroperitoneal adipose tissues were rapidly removed and weighed, frozen in liquid nitrogen, and stored at −80°C until RNA analysis.

### Measurement of Circulating Parameters (Glucose and Free Fatty Acid)

Blood glucose concentration was measured using an Accu-Chek Glucometer (Roche Diagnostics, Barcelona, Spain) in blood obtained from the neck at the moment of sacrifice. Non-esterified free fatty acids (NEFA) levels were measured in serum using an enzymatic colorimetric NEFA-HR2 kit (from WAKO, Neuss, Germany).

### Histological Analysis

tPVAT and IAT samples were fixed by immersion in 4% paraformaldehyde in 0.1 M sodium phosphate buffer, pH 7.4, overnight at 4°C, washed in phosphate buffer, dehydrated in a graded series of ethanol, cleared in xylene and embedded in paraffin blocks for light microscopy. Five-micrometer-thick sections of tissue were cut with a microtome and mounted in slides. The area of white adipocytes was measured in hematoxylin/eosin stained section. Images from light microscopy were digitalized and the area of at least 200 cells of each section was determined using Axion Vision Software (Carl Zeiss, S.A., Barcelona, Spain).

### Immunohistochemistry Analysis of UCP1 in Aortic Perivascular and Inguinal Adipose Tissue

Five-micrometers sections of adipose tissue of the different experimental groups were immunostained by means of the avidin-biotin technique (Hsu et al., 1981). Briefly, tissue sections were incubated with 5% H2O2 in water for 15 min to block endogenous peroxidase and then with normal goat serum 2% in PBS pH 7.3 to block unspecific sites and then overnight at 4°C with primary rabbit polyclonal UCP1 antibody (GeneTex International Corporation, CA, United States) diluted 1:370 in PBS overnight at 4°C. The primary antibody that was used cross-reacts with rat UCP1, and has been previously validated for ferrets (Fuster et al., 2009; Sánchez et al., 2009). Sections were then incubated with the corresponding biotinylated anti-rabbit IgG secondary antibody (Vector Laboratories, Burlingame, CA, United States), diluted 1:200, and finally with ABC complex (Vectastain ABC kit, Vector, CA, United States). Peroxidase activity was revealed with Sigma Fast 3,3’-diaminobenzidine (Sigma-Aldrich, St. Louis, MO, United States) as substrate. Finally, sections were counterstained with hematoxylin and mounted in Eukitt (Kindler, Freiburg, Germany). Images were acquired with a Zeiss Axioskop 2 microscope equipped with AxioCam ICC3 digital camera and AxioVision 40V 4.6.3.0 Software (Carl Zeiss, S.A., Barcelona, Spain). White adipocytes, cells of the aorta, lymph nodes and nerves were not stained, confirming the specificity of the staining for multilocular/brite adipocytes.

### Total RNA Isolation

Total RNA from tPVAT and IAT samples was extracted using Tripure Reagent (Roche Diagnostics, Barcelona, Spain). RNA samples were purified with E.Z.N.A. MicroElute RNA Clean Up (Omega Bio-tek, VT, United States), and with 3M sodium acetate and absolute ethanol. RNA yield was quantified using a NanoDrop ND 1000 spectrophotometer (NanoDrop Technologies, Wilmington, DE, United States) and the integrity was measured on an Agilent 2100 Bioanalyzer with RNA 6000 Nano chips (Agilent Technologies, South Queensferry, United Kingdom).

### Microarray Processing

A whole genome ferret-specific gene expression microarray was designed by the Genomics and Translational Genetics Service of the Príncipe Felipe Research Center (Valencia, Spain). The microarray was designed as a 2 × 400 k G4861A (AMADID-064079) Agilent array (Agilent Technologies, Inc., Santa Clara, CA, United States).

For the microarray analysis, we used tPVAT and IAT RNA samples of control (*n* = 7) and cold-exposed ferrets (*n* = 6). For microarray hybridation 0.2 μg RNA of each sample was reverse transcribed using the Agilent Low Input Quick Amp Labeling kit (Agilent), according to the manufacturer’s protocol, with some modifications (all materials and reagents were from Agilent Technologies, Palo Alto, CA, United States). Half of the cDNA sample (5 μl) was used for linear RNA amplification and labeling with Cy5 or Cy3. All reactions were performed using half of the amounts indicated by the manufacturer (as previously described in van Schothorst et al., 2007). Briefly, a transcription master mix was prepared (0.375 μl nuclease-free water; 1.6 μl 5 X Transcription buffer; 0.3 μl 0.1 M DTT; 0.5 μl NTP mix; 0.115 μl T7 RNA Polymerase Blend and 0.12 cyanine 3-CTP or cyanine 5-CTP per sample) and added to 5 μl cDNA. *In vitro* transcription and labeling were carried out at 40°C for 2 h. The labeled cRNA samples were purified using Qiagen Rneasy minispin columns (Qiagen, Venlo, Netherlands). Dye incorporation and cRNA concentration was measured using the microarray measurement of the Nanodrop spectrophotometer (NanoDrop Technologies). Yield of each individual sample was at least 1.875 ng and specific activity 6.0 pmol Cy3 or Cy5 per μg cRNA. All approved high quality Cy5 cRNA’s were pooled to serve as standard reference pool; the pool consisted of RNA isolated from different tissues to increase signal distribution over all probes. This pool included 10 samples from peripheral blood mononuclear cells samples of control and cold-exposed ferrets. Hybridization was performed by preparing a cRNA target solution containing 1.875 ng Cy3-labeled cRNA, 1.875 ng Cy5-labeled pool cRNA, 25 μl of 10X blocking agent, 5 μl 25X Fragmentation Buffer in a total volume of 125 μl. The samples were incubated at 60°C for 30 min, and then immediately cooled on ice for 1 min. After that, 125 μl 2x GEx hybridization buffer HI-RPM was added and hybridized on the Agilent arrays (Agilent Technologies, Inc., Santa Clara, CA, United States), for 17 h at 65°C in Agilent hybridization chambers in an Agilent hybridization oven rotating at 10 rpm. After hybridization, the arrays were subsequently washed with GE wash buffer 1, for 1 min at room temperature, and GE wash buffer 2, for 1 min at approximately 37°C, according to manufacturer’s protocol (Agilent Technologies).

### Normalization and Transcriptome Data Analysis

The arrays were scanned with an Agilent Microarray Scanner (Agilent Technologies Scanner G2505C, Agilent Technologies) with a Profile AgilentG3_GX_2Color. Median density values and background values of each spot were extracted for both the experimental samples (Cy3) and the reference samples (Cy5). Quality control for every microarray was performed visually by using ‘Quality control graphs’ from Feature extraction and M-A plots and box plots, which were made using limmaGUI in R (Wettenhall and Smyth, 2004). Data were imported into GeneMaths XT 2.12 (Applied Mathematics, Sint-Martens-Latem, Belgium) for background correction and normalization. Target signals with an average intensity twice above the background were selected to increase accuracy of the data. Normalization and data analysis were performed as published (Pellis et al., 2003). In addition to the set of 26 microarray from adipose tissue samples, 10 microarray from peripheral blood cells, performed at the same time, were also included for normalization. Blood samples data analyses are not included in the present work. After normalization procedure, sequences were averaged per unique sequence (45,328 sequences of 300,577 detected probes, which corresponded to 19,282 genes). To select unique genes we considered those sequences with higher statistical differences between the cold vs. control group of IAT assessed by Student’s *t*-test in GeneMaths XT. Statistical differences in tPVAT between the cold and control groups was also assessed by Student’s *t-*test in GeneMaths XT, and statistical differences between tPVAT and IAT were assessed by Student’s *t*-test in Microsoft Excel; the generated *p*-values were used to obtain insight into significantly affected genes. Fold change calculations were performed in Microsoft Excel. In order to compare gene expression pattern at control temperature of the two studied adipose tissues, we selected the genes with fold change > 2, using a significance threshold of *p* < 0.01. For analysis of the effect of cold exposure, genes with the highest or the lowest fold change, with a threshold of *p* < 0.05, were selected from both tissues. Moreover, all sequences were used to perform pathway analysis using MetaCore^TM^ (GeneGo, St. Joseph, MI, United States) to determine the effect of cold exposure. In addition, the top 50 up- and down-regulated genes were manually classified into biological processes using available databases (Genecards, NCBI, WikiPathways, PubMed). Microarray data has been deposited in NCBI Gene Expression Omnibus (GEO) under accession number GSE62353 for tPVAT and GSE62351 for IAT datasets.

Volcano plots of the microarray data were made using GraphPad Prism version 6 (Graphpad Software, San Diego, CA, United States). Principal compound analysis was made using SPSS for windows (version 15.0; SPSS, Chicago, IL, United States).

### Reverse Transcription Quantitative Real-Time Polymerase Chain Reaction (RT-qPCR) Analysis

To validate microarray data for the IAT samples we analyzed mRNA expression of selected genes by RT-qPCR. The microarray confirmation for tPVAT microarray data was previously reported (Reynés et al., 2017). In IAT the following genes were analyzed: *Col3a1*, *Cpt2*, *Fabp3*, *Idh3b*, *Lum*, *Sucla2*, and *Ucp3*. These genes were selected based on their relevant biological function and/or because they are included in the top regulated genes. Fifty ng of total RNA from IAT was reverse transcribed to cDNA using iScript cDNA synthesis kit (BIO-RAD, Madrid, Spain) at 25°C for 5 min, 42°C for 30 min, and 85°C for 5 min, in an Applied Biosystems 2720 Thermal Cycler (Applied Biosystems, Madrid, Spain). Each PCR was performed from diluted (1/10) cDNA template, forward and reverse primers (5 μM), and Power SYBER Green PCR Master Mix (Applied Biosystems) in a total volume of 11 μl, with the following profile: 10 min at 95°C, followed by a total of 40 temperature cycles (15 s at 95°C and 1 min at 60°C). In order to verify the purity of the products, a melting curve was produced after each run according to the manufacturer’s instructions. The threshold cycle (Ct) was calculated using the instrument’s software (StepOne Software v2.0, from Applied Biosystems) and the relative expression of each mRNA was calculated as a percentage of controls rats, using the Pfaffl’s method (Pfaffl, 2001).

For the genes used to validate the microarray analysis, data were normalized against the reference gene Apolipoprotein O (*Apoo*). *Apoo* was chosen because our microarray data showed equal expression over all microarrays in control and cold-exposed ferrets. Primers for the different genes are described in Table 1 and were obtained from Sigma Genosys (Sigma-Aldrich Química SA, Madrid, Spain).

**TABLE 1:** Nucleotide sequences of primers and amplicon size used for RT-qPCR amplification.

**Gene**	**Forward primer (5′–3′)**	**Reverse primer (5′–3′)**	**Amplicon size (bp)**
*Col3a1*	GCTGTTGAGGGAGGATGTT	ATTAGGAGGACGAGGAGGAG	222
*Cpt2*	CATTCAACCCTGACCCAAAG	AGGAAGGCACAAAGCGTATG	186
*Fabp3*	CTCGGTGTGGGTTTTGCTAC	ACGGTGGACTTGACCTTCCT	185
*Idh3b*	GGGAGCAGACAGAAGGAGAA	GGAACAATCCATCCCCAAG	198
*Lum*	GCCTATTTCATCACAAGCACAG	CCCATTCTTTTTGGCACATT	186
*Sucla2*	CAGGAAGATGAAAGGGAGAAA	TCTGTTACTTGATGGACTGTGG	191
*Ucp3*	GCGAGCAACAGGAAATACAG	CAAAGGCAGAGATGAAGTGG	217
*Apoo* (Reference gene)	TGGTGTTATCGGTTTTGCTG	CTTCACATTTCCTGGCTTTTG	235

### Statistical Analysis

Data of body weight, adiposity, serum parameters and the confirmatory results of the microarray data are expressed as the mean ± SEM. Differences between groups were analyzed using Student’s *t*-test. All analyses were performed with SPSS for windows (version 15.0; SPSS, Chicago, IL, United States). Threshold of significance was defined at *p*-value < 0.05. The statistical analysis of the microarray data has been indicated in the microarray data analysis section.

## Results

### Cold Exposure Induced Fat Mass Mobilization in Ferrets

As we previously reported using the same set of animals (Reynés et al., 2017), cold exposure decreased the mass of the inguinal, retroperitoneal and interscapular white adipose tissues in cold-exposed ferrets (58% decrease in the IAT, and 74% decrease both in the interscapular and retroperitoneal depots). This lower fat mass content was not translated into a statistically significant lower body weight, however, these animals attained a 16% lower body weight gain compared to control animals during the week in which they were exposed to 4°C, in accord with the lower adiposity. Specifically, animals of the cold-exposed group weighed 559 ± 31 g before and 510 ± 36 g after cold exposure; while those of the control group weighed 581 ± 51 g and 623 ± 60 g, before and after this week, respectively. Moreover, cold exposure induced an increase in circulating NEFA levels (0.955 ± 0.078 mM in cold-exposed animals vs. 0.619 ± 0.085 mM in the control group, Student’s *t*-test, *p* < 0.05). Circulating glucose levels were also analyzed, but they were not affected by cold acclimation (data not shown).

### Cold Exposure Induced the Appearance of UCP1-Positive Adipocytes in tPVAT

**FIGURE 1:**
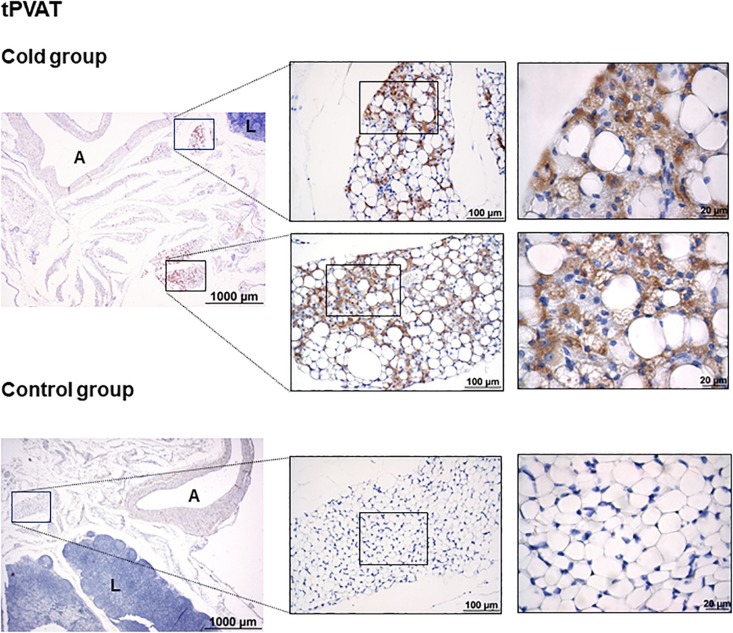
Uncoupling protein 1 immunostaining in tPVAT of ferrets acclimatized to different room temperatures: 22°C (control group) or 4°C (cold group) for 1 week. A strong browning induction in cold-exposed ferrets is observed, characterized by increased appearance of adipocytes positive for UCP1 protein. A representative image of an animal of each group is represented. For each image, magnifications of 25×, 200×, and 630× are shown. A, aorta; L, lymph node.

**FIGURE 2:**
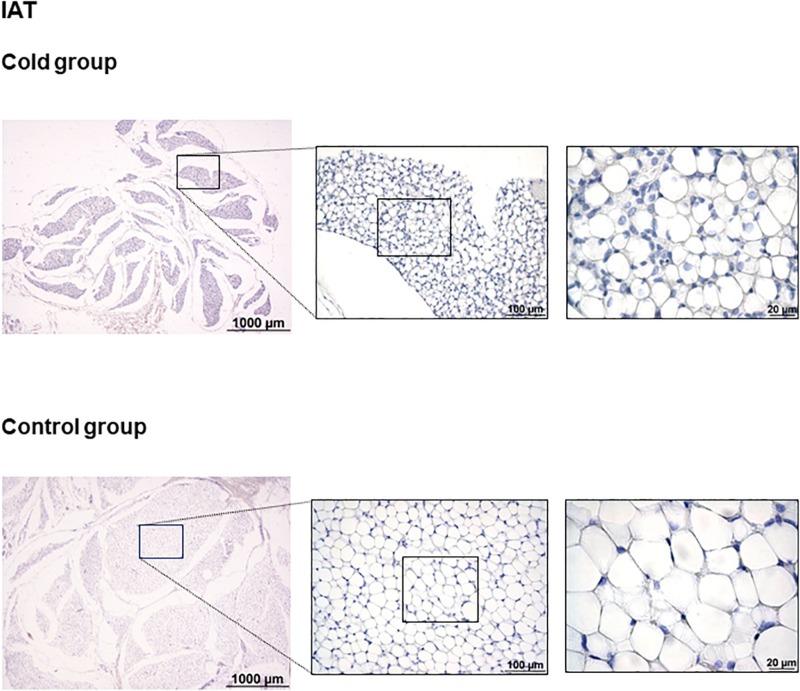
Uncoupling protein 1 immunostaining in IAT of ferrets acclimatized to different room temperatures: 22°C (control group) or 4°C (cold group) for 1 week. No appearance of UCP1 positive cells was observed as result of cold exposure. A representative image of an animal of each group is represented. For each image, magnifications of 25×, 200×, and 630× are shown.

As shown in Figure 1, immunohistochemical analysis revealed that cold exposure induced a remodeling of the tPVAT, with the appearance of multilocular adipocytes highly stained for UCP1 in sparse lobules within the adipose depot. This is similar to what occurred in the retroperitoneal adipose tissue, which, according to our previous data, is a depot with a high degree of browning in response to proper stimuli, such as cold or diet (Fuster et al., 2009; Sánchez et al., 2009). Interestingly, cold-induced browning was not observed in the IAT (Figure 2); the complete area of the different sections was analyzed. In spite of the lower mass of the IAT, morphometric analysis performed in this tissue did not show a significant smaller cell size of the unilocular adipocytes as result of cold exposure (331 ± 68 vs. 497 ± 54 μm^2^, in cold-exposed and control ferrets, respectively; Student’s *t*-test, *p*-value < 0.1).

### Differential Gene Expression Pattern Between tPVAT and IAT Was Diminished as Result of Cold Exposure

**FIGURE 3:**
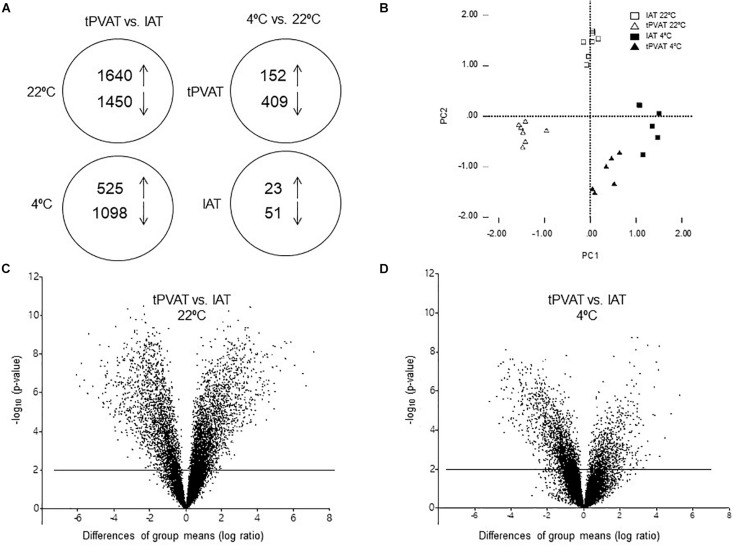
**(A)** Schematic overview of regulated genes in tPVAT and in IAT in control (22°C) vs. cold (4°C) exposed ferrets (Student’s *t*-test, *p*-value < 0.01, with an absolute fold change ≥ 2). The arrows show the number of genes up- and down-regulated in each analysis. **(B)** PCA analysis of gene expression in tPVAT and IAT at 22 and 4°C of a subset of genes being differentially expressed in tPVAT vs. IAT at control (22°C) temperature (Student’s *t*-test, *p*-value < 0.01, with an absolute fold change ≥ 2). Volcano plot for all expressed probes in control **(C)** and cold conditions **(D)**. Minus log10 *p*-value of all genes are plotted against the differences of group means (log ratio) of each gene, tPVAT vs. IAT. The line indicates the threshold *p*-value < 0.01. The results represent data from the control (*n* = 7) and cold (*n* = 6–7) groups.

To gain a global view of the molecular basis of biologic differences between tPVAT and IAT in ferrets, we performed a whole genome transcriptome analysis. A total of 19,282 genes were expressed, of which 3,090, representing 16%, being differentially expressed between tPVAT and IAT (Student’s *t*-test, *p*-value < 0.01, with an absolute fold change ≥ 2). Expression of 1,640 genes was higher and of 1,450 lower in tPVAT vs. IAT (Figure 3A). One week of exposure to 4°C decreased the gene expression differences between both adipose tissue depots to 1,623 genes, equaling 8%; with only 525 genes being higher and 1,098 lower expressed in tPVAT vs. IAT (Figure 3A). Volcano plots clearly show how the differences between both adipose tissue depots were reduced by cold exposure (Figures 3C,D). This is also observed by PCA analysis (Figure 3B), which also shows that tPVAT and IAT at 22°C have indeed a clearly different gene expression profile, which becomes more similar profile due to cold exposure.

In spite of the importance of UCP1 in adaptive thermogenesis and of the increase of UCP1-positive stained adipocytes in tPVAT of cold-exposed animals, its mRNA expression was not altered –as measured by microarray analysis– by cold exposure in tPVAT and IAT. Therefore, we focused our attention on a battery of relevant genes known as brown adipocyte markers or involved in the thermogenic response (see Table 2). Interestingly, most of these genes, such as *Cidea*, *Ebf3*, *Fabp3*, *Ppara*, and *Ppargc1a* had a higher expression in IAT than in tPVAT in control and cold-exposed animals, suggesting a brite nature of IAT. However, cold acclimation particularly increased the expression in tPVAT of *Ebf3*, *Fapb4*, *Fndc5*, *Pdk4* and *Ppargc1b*, known brown/thermogenic markers.

**TABLE 2:** Comparison of gene expression of brown/brite adipocyte markers in tPVAT vs. IAT and in cold vs. control temperature (Student’s *t*-test).

	**tPVAT vs. IAT**				**4°C vs. 22°C**			
		
	**22°C**		**4°C**		**IAT**		**tPVAT**	
				
**Gene symbol**	***p*-value**	**Fold change**	***p*-value**	**Fold change**	***p*-value**	**Fold change**	***p*-value**	**Fold change**
*Cidea*	**< 0.001**	**−8.98**	**< 0.001**	**−11.0**	**< 0.05**	**+ 1.69**	0.35	+ 1.38
*Ebf3*	**< 0.001**	**−4.60**	**< 0.001**	**−3.11**	0.45	–1.12	**< 0.05**	**+ 1.32**
*Fabp3*	**< 0.001**	**−5.84**	**< 0.001**	**−8.87**	**< 0.05**	**+ 3.10**	0.12	+ 2.04
*Fgf21*	0.68	+ 1.09	0.38	–1.19	0.92	–1.02	0.16	–1.33
*Fndc5*	**< 0.001**	**−1.97**	0.08	+ 1.49	**< 0.01**	**−1.95**	**< 0.05**	**+ 1.50**
*Pdk4*	**< 0.01**	**−5.85**	0.22	–1.79	0.17	+ 1.75	**< 0.01**	**+ 5.73**
*Ppara*	**< 0.001**	−**4.03**	**< 0.01**	**−2.69**	0.83	+ 1.03	0.08	+ 1.55
*Ppargc1a*	**< 0.001**	**−4.49**	**< 0.001**	**−4.76**	0.16	+ 1.36	0.30	+ 1.28
*Ppargc1b*	**< 0.001**	**−2.98**	<0.05	–1.81	0.56	–1.13	**< 0.05**	**+ 1.46**
*Prdm16*	0.53	+ 1.14	0.91	–1.02	0.67	+ 1.10	0.73	–1.07
*Tbx15*	0.30	–1.25	0.61	–1.23	0.91	+ 1.03	0.90	+ 1.04
*Ucp1*	0.92	–1.02	0.81	–1.20	0.13	+ 1.78	0.50	+ 1.51

**p*-value is indicated. Fold change: + indicates up-regulated and –indicates down-regulated genes. Bold indicates statistical significance. The results represent data from the control (*n* = 7) and cold (*n* = 6–7) groups.*

### Cold Exposure Induced a Higher Gene Expression Response in tPVAT Than in IAT

**FIGURE 4:**
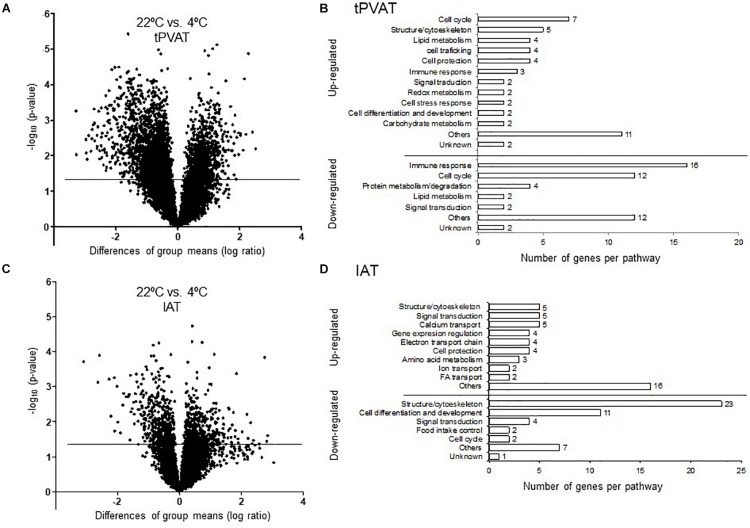
Volcano Plot for all expressed probes by microarray analysis in tPVAT **(A)** and IAT **(C)**. Minus log10 *p*-value of all genes are plotted against the differences of group means (log ratio) of each gene, cold vs. control. The line indicates the threshold *p*-value < 0.05. Detailed manual classification of the 50 top up- and down-regulated genes based on their fold change (Student’s *t*-test, *p*-value < 0.05) in tPVAT **(B)** and in IAT **(D)**. The results represent data from the control (*n* = 7) and cold (*n* = 6–7) groups.

Our microarray data show that cold exposure produced a higher impact on gene expression in tPVAT than in IAT. Thus, 561 in tPVAT vs. 74 in IAT from a total of 19,282 genes changed expression in the cold condition (Figure 3A). The impact of cold exposure on transcriptional changes is illustrated in Figures 4A,C using Volcano Plots, which shows that tPVAT is primarily affected.

**FIGURE 5:**
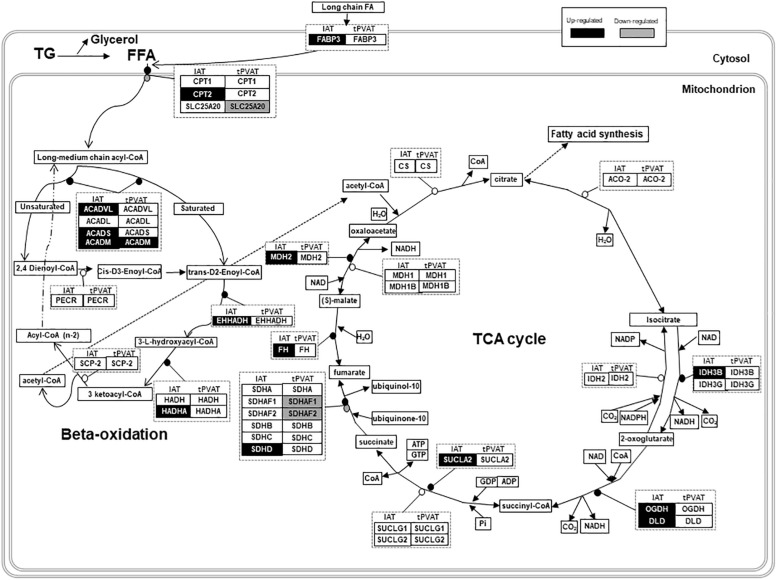
Schematic overview of gene expression regulation of fatty acid oxidation and tricarboxylic acid (TCA) cycle in tPVAT and IAT. Up-regulated genes are marked in black and down-regulated are marked in gray (Student’s *t*-test, *p*-value < 0.05). Source: WikiPathways (adapted). According to the license terms of WikiPathways, users are free to use the pathway images in presentations, documents, websites and publications; as well as to use the pathway data in analyses, qualitative or quantitative, while citing or giving appropriate attribution (https://www.wikipathways.org/index.php/WikiPathways:License_Terms). ACADL, Acyl-CoA Dehydrogenase, Long Chain; ACADM, Acyl-CoA Dehydrogenase, C-4 To C-12 Straight Chain; ACADS, Acyl-CoA Dehydrogenase, C-2 To C-3 Short Chain; ACADVL, Acyl-CoA Dehydrogenase, Very Long Chain; ACO-2, Aconitase; CPT1, Carnitine Palmitoyltransferase 1; CPT2, Carnitine Palmitoyltransferase 2; CS, Citrate Synthase; DLD, Dihydrolipoamide Dehydrogenase; EHHADH, Enoyl-CoA Hydratase And 3-Hydroxyacyl CoA; FFA, Free Fatty Acids; FABP3, Fatty Acid Binding Protein 3; FH, Fumarate Hydratase; HADH, Hydroxyacyl-CoA Dehydrogenase; HADHA, Hydroxyacyl-CoA Dehydrogenase/3-Ketoacyl-CoA; IDH2, Isocitrate Dehydrogenase (NADP(+)) 2, Mitochondrial; IDH3B, Isocitrate Dehydrogenase 3 (NAD(+)) Beta; IDH3G, Isocitrate Dehydrogenase 3 (NAD(+)) Gamma; MDH1, Malate Dehydrogenase 1; MDH1B, Malate Dehydrogenase 1B; MDH2, Malate Dehydrogenase 2; NAD, Nicotinamide Adenine Dinucleotide; OGDH, Oxoglutarate Dehydrogenase; PECR, Peroxisomal *Trans*-2-Enoyl-CoA Reductase; SCP-2, Sterol Carrier Protein 2; SDHA, Succinate Dehydrogenase Complex Flavoprotein Subunit A; SDHAF1, Succinate Dehydrogenase Complex Assembly Factor 1; SDHAF2, Succinate Dehydrogenase Complex Assembly Factor 2; SDHB, Succinate Dehydrogenase Complex Iron Sulfur Subunit B; SDHC, Succinate Dehydrogenase Complex Subunit C; SDHD, Succinate Dehydrogenase Complex Subunit D; SLC25A20, Solute Carrier Family 25 Member 20; SUCLA2, Succinate-CoA Ligase ADP-Forming Beta Subunit; SUCLG1, Succinate-CoA Ligase Alpha Subunit; SUCLG2, Succinate-CoA Ligase GDP-Forming Beta Subunit; TG, Triglycerides.

To obtain a more detailed view on the impact of cold exposure, we manually classified the top 50 up- and down-regulated genes of each tissue into different metabolic pathways (Student’s *t*-test, *p*-value < 0.05) (Supplementary Tables). Results, represented in Figures 4B,D, show that cold exposure induced different gene expression adaptations in tPVAT compared to IAT. In tPVAT, up-regulated genes as result of cold exposure were involved in different metabolic pathways, while the down-regulated genes were mainly involved in immune response and cell cycle (Figure 4B). For IAT, the top up-regulated genes were also involved in many different processes; however, the top down-regulated genes were mainly genes involved in structure/cytoskeleton organization, and in cell differentiation and development (Figure 4D). Additionally, a MetaCore pathway analysis was performed taking all probes significantly affected by cold exposure into account (Student’s *t*-test, *p* < 0.05 for cold vs. control). Table 3 shows the top 10 pathways affected in each adipose tissue. In tPVAT the top regulated pathways were cell cycle, cell differentiation and development, immune response, protein metabolism and transcriptional regulation (Table 3A), coincident with the manual classification (Figure 4B). In the IAT, the most affected pathways were cell adhesion, cytoskeleton, immune response, energy metabolism and muscle contraction (Table 3B). We were particularly interested in the differential response observed for energy metabolism (including tricarboxylic acid –TCA– cycle and fatty acid beta-oxidation), which appeared as one of the affected pathways in response to cold exposure in the IAT but not in the tPVAT. In order to further study this differential response we manually compared the expression of genes involved in the tricarboxylic acid cycle and in fatty acid beta-oxidation in both adipose tissue depots. Our results show that most of the up-regulated genes in IAT were down-regulated or not affected in tPVAT (Figure 5). A more detailed interpretation of these data can be found in the discussion section.

**TABLE 3:** Top 10 regulated pathways analyzed by MetaCore^TM^ in the tPVAT **(A)** and IAT **(B)**.

**Top 10 pathways**	***p*-value**

**(A) tPVAT**	
**Cell cycle**	
DNA damage ATM/ATR regulation of G1/S checkpoint	6.05E-09
Role of APC in cell cycle regulation	6.15E-10
Spindle assembly and chromosome separation	9.94E-12
Start of DNA replication in early S phase	4.37E-12
The petaphase checkpoint	7.02E-13
Transition and termination of DNA replication	3.67E-07
**Cell differentiation and development**	
Development WNT signaling pathway	7.48E-08
**Immune response**	
Inhibitory PD-1 signaling in T cells	3.93E-07
**Metabolism**	
Protein folding and maduration POMC processing	1.08E-11
**Transcriptional regulation**	
Transcriptional epigenetic regulation of gene expression	6.42E-08

**(B) IAT**	

**Cell adhesion**	
Integrin-mediated cell adhesion and migration	1.90E-04
**Cytoskeleton**	
Cytoskeleton remodeling	6.75E-04
Regulation of actin cytoskeleton by Rho GTPases	9.01E-04
Role of PKA in cytoskeleton reorganization	3.52E-04
**Immune response**	
Platelet activating factor/PTAFR pathway signaling	5.00E-04
**Energy metabolism**	
Lysine metabolism	1.54E-04
Mitochondrial unsaturated fatty acid beta-oxidation	7.41E-04
Propionate metabolism	2.99E-04
Tricarboxylic acid cycle	2.94E-04
**Muscle contraction**	
Relaxing signaling pathway	1.10E-03

*Regulated pathways were classified into: cell cycle, cell differentiation and development, immune response, metabolism and transcriptional regulation in the tPVAT; and cell adhesion, cytoskeleton, immune response, energy metabolism and muscle contraction in the IAT; *p*-values are given.*

### Confirmation of Microarray Data Results by RT-qPCR

Real-time polymerase chain reaction was performed on total RNA from IAT samples in order to confirm the microarray data; the tPVAT microarray confirmation was previously reported (Reynés et al., 2017). We selected 7 genes, 2 involved in the TCA cycle: *Idh3b* and *Sucla2*; 2 involved in cytoskeleton organization and cell adhesion: *Col3a1* and *Lum*; 2 involved in fatty acid metabolism and transport: *Cpt2* and *Fabp3;* and *Ucp3*, which has been proposed as a potential alternative uncoupler. RT-qPCR confirmed the microarray data for all genes analyzed (Table 4).

**TABLE 4:** Real-time polymerase chain reaction confirmation of microarray data.

**Gene symbol**	**Gene name**	**Sequence ID**	**Microarray**		**RT-qPCR**	
				
			**FC**	***p*-value**	**FC**	***p*-value**
*Col3a1*	Collagen, Type III, Alpha 1	XM_004763363.1	−8.58	0.0002	−1.70	0.0050
*Cpt2*	Carnitine palmitoyl transferase 2	XM_004774429.1	+ 1.42	0.0300	+ 1.48	0.0054
*Fabp3*	Fatty acid binding protein 3, muscle and heart	XM_004740951.1	+ 3.10	0.0157	+ 4.98	0.0017
*Idh3b*	Isocitrate dehydrogenase 3 (NAD +) beta	XM_004772915.1	+ 1.58	0.0060	+ 3.31	0.0000
*Lum*	Lumican	XM_004748555.1	−6.00	0.0001	−1.90	0.0026
*Sucla2*	Cuccinate-CoA ligase, ADP-forming, beta subunit	XM_004759183.1	+ 1.69	0.0090	+ 1.55	0.0103
*Ucp3*	Uncoupling protein 3	XM_004768064.1	+ 3.03	0.0044	+ 4.18	0.0020

*Fold change (FC), + indicates up-regulated and – indicates down-regulated genes. *p*-values of microarray and RT-qPCR data are given (Student’s *t*-test). The results represent data from the control (*n* = 7) and cold (*n* = 6–7) groups.*

## Discussion

Perivascular white adipose tissue in humans exerts a paracrine-based protective action, which changes to a detrimental role in the pathogenesis of obesity and metabolic syndrome, contributing to perivascular inflammation and, thus, to cardiovascular risk (Payne et al., 2012; Mazurek and Opolski, 2015; Aldiss et al., 2017). However, PVAT browning has been linked to a cardioprotection (Aldiss et al., 2017; Xiong et al., 2017; Villarroya et al., 2018), in relation to a decrease in pro-inflammatory signals, associated with a BAT-like phenotype in PVAT (Villarroya et al., 2018). Human tPVAT is a white adipose tissue, which possesses some brown adipocyte features, such as expression of brown-adipocyte specific genes (Chatterjee et al., 2009). This is in contrast to what is described in rodents, classically used as models to study thermogenesis, in which tPVAT presents a gene expression pattern and possess a morphology closer to BAT (Fitzgibbons et al., 2011; Tran et al., 2018). Previous studies of our group suggested that ferrets could be a useful animal model for thermogenesis and browning studies, as their adipose tissue is closer to that of humans (Fuster et al., 2009). As in humans, ferrets do not possess a well-defined BAT, but white adipose tissue depots with scarce brown-like adipocytes that increase in response to thermogenic stimuli, such as cold or dietary factors (Fuster et al., 2009; Sánchez et al., 2009). Due to the importance of PVAT browning for cardiovascular health, we have here studied the thoracic aortic PVAT of ferrets and its response to cold acclimation in detail. We focused on the thoracic region of the aorta because is the one with the greater thermogenic capacity reported in humans and rodents (Chatterjee et al., 2009; Fitzgibbons et al., 2011; Tran et al., 2018). In our study, we have compared tPVAT to the inguinal subcutaneous adipose tissue, the adipose tissue depot with a higher browning potential in rodents (Wang and Yang, 2017), for perspective and to obtain insight in tissue-specific adaptations to a low ambient temperature.

Cold exposure recruits and activates BAT, and induces browning in WAT depots (Palou et al., 1998; Bonet et al., 2013). According to our data, no browning is observed in the immunohistochemical analysis upon cold acclimation in the IAT. This is in sharp contrast with tPVAT, where cold exposure induces evident browning, with the appearance of multilocular brite adipocytes intensely stained for UCP1, suggesting an increased thermogenic capacity in this adipose tissue depot in ferrets. This would be probably related to the physiological need to maintain blood temperature at systemic level. This relevant tPVAT browning is similar to that previously reported by our group in the retroperitoneal adipose tissue of ferrets, in which multilocular UCP1-possitive adipocytes appeared and UCP1 protein levels increased in response to cold exposure (Fuster et al., 2009) or to nutritional treatment (Sánchez et al., 2009).

Global transcriptome analysis, performed in the tPVAT and IAT, revealed a substantial difference in gene expression between both adipose depots, with 16% of the genes differentially expressed in animals acclimated to control temperature. However, this gene expression difference was ameliorated to 8% when comparing both adipose depots in cold-exposed animals. The closer resemblance in the cold between both adipose depots is in part due to the induction of browning in tPVAT. Already at 22°C IAT expressed higher levels than tPVAT of key brown/brite markers, showing a more noticeable brite signature. However, cold exposure increased mRNA expression of several markers, such as *Ebf3*, *Fapb4*, *Fndc5*, *Pdk4* and *Ppargc1b*, specifically in the tPVAT, which appeared more sensitive to browning induction in response to cold, as evidenced by immunohistochemical analysis that showed a prominent appearance of UCP1-positive brite cells in this tissue. Increased similarity between tPVAT and IAT in cold-exposed animals was also due to convergence of gene expression. For example, mRNA levels of *Fndc5*, a gene coding for the precursor of irisin, which is a browning inducer (Boström et al., 2012), decreased because of cold exposure in the IAT but increased in the tPVAT. However, the most highly affected pathway in tPVAT was not related to lipid metabolism, but to immune response, with immune-related genes as the most down-regulated in this depot. A down-regulation of immune response could be explained by its energy demanding nature (French et al., 2009). We previously proposed that the cold exposure-induced immunosuppression in tPVAT, also evidenced in immune cells, could modulate vascular inflammation decreasing cardiovascular risk (Reynés et al., 2017), a notion that was recently confirmed in rats (Li et al., 2017). In addition to down-regulation of immune response genes in tPVAT, using specific cold sensitive transgenic mice, it was shown that PVAT-dependent thermogenesis prevented atherosclerosis development (Xiong et al., 2017). Thus, our ferret data suggest that the increased browning and decreased inflammation in tPVAT may have a functional cardioprotective role. This assumption is supported by the concept that inflammatory adipocyte-derived factors from a dysfunctional PVAT have been related to cardiovascular risk (Nosalski and Guzik, 2017). Therefore, a lower fat mass (due to browning induction and/or increased white adipose tissue lipolysis), as well as immune-suppression, would be related to a lower production of inflammatory signals acting on vascular endothelia with a consequent protection from vascular inflammation, cumulatively having a protective role.

Besides thermogenic stimulation, it is widely known that cold acclimation induces an increased metabolic rate, evidenced by enhanced lipid catabolism (lipolysis and beta-oxidation), which occurs especially in WAT, providing increased serum NEFA levels (Trayhurn, 2005). In fact, MetaCore pathway analysis performed using all the differentially expressed genes (*p*-value < 0.05) revealed that energy metabolism, including TCA cycle and fatty acid beta-oxidation, increased in IAT of the cold group, a pattern which was not so clearly evident in tPVAT (see scheme in Figure 5). Thus, in IAT, cold exposure up-regulated genes involved in beta-oxidation (*Acadm*, *Acads, Acadvl, Cpt2*, *Ehhadh* and *Hadha*), and in the TCA cycle (*Dld*, *Fh, Idh3b*, *Mdh2, Ogdh*, *Sucla2* and *Sdhd*). Related to this, between the top up-regulated genes as result of cold acclimation in the IAT, we also found genes involved in long chain fatty acid transport into the adipocytes (*Fabp3*), and genes of the mitochondrial electron transport chain (such as cytochrome c complex) and oxidative phosphorylation (*Slc25a4*). Altogether, these cold exposure adaptations reflect an increased use of fatty acids in IAT, which is not so evident in tPVAT.

Notably, associated with the increased capacity for lipid catabolism observed in IAT, cold acclimation induced a lower mass of the whole depot. Interestingly, it has been reported, in rodents, that during adaptation to cold, and in connection to the decreased adipocyte size resulting from increased lipolysis, there is an expansion of extracellular space (Suter, 1969). Our data in ferret IAT go in the same direction, as 23 of the 50 down-regulated genes by cold exposure were related to cell structure and cytoskeleton organization, reflecting structural adaptations in this tissue and resembling adipose tissue adaptations as seen after massive weight loss (Roumans et al., 2017; Vink et al., 2017). Particularly, 32% of these down-regulated genes encoded for collagen or collagen-related pathways. Two of these are *Col6a3* and *Lum* that, besides their structure-related functions, have also been related with obesity development (Takahashi et al., 1993; Nakajima et al., 1998; Khan et al., 2009). *Col6a3* encodes a collagen type highly expressed in WAT that possesses an important role in adipocyte physiology and is positively correlated with the dysregulation of human adipose tissue in obesity (Khan et al., 2009). Absence of *Col6a3* is associated with lower body weight and fat mass, and with an improved metabolic phenotype (Nakajima et al., 1998; Khan et al., 2009). *Lum*, which encodes lumican, a proteoglycan identified to bind fibrillar collagen VI, is positively correlated with obesity (Khan et al., 2009). Moreover, interestingly, it has been recently reported that reduced adipose tissue fibrosis mediated by a cold-inducible transcription factor improves glucose homeostasis with independence of UCP1-mediated thermogenesis (Hasegawa et al., 2018). Thus, taken together, our results suggest that the reduction of IAT mass in cold-exposed animals is accompanied with a decreased presence of extracellular matrix components, as collagen and the proteoglycans, which could be a signal of an improved metabolic state because of cold exposure.

We conclude that acclimation to cold reduces the gene expression differences between tPVAT and IAT, with both adipose tissues responding to cold exposure in a depot-specific manner. Thoracic aortic PVAT is particularly sensitive to cold exposure, as evidenced by a higher gene expression response of brown/brite markers in comparison to the IAT, and by the dramatic morphologic remodeling of the tissue, with the appearance of abundant UCP1-positive stained brite adipocytes. Moreover, cold exposure induced a drastic immunosuppression in this PVAT depot, which is considered to contribute to an improved cardiovascular health. On the other hand, the IAT follows a different cold-exposure adaptation pattern, not only related to increased browning, but with increased fatty acid mobilization as well as an increased TCA cycle, which is translated into lower mass of this adipose depot. This is not coincident with what is reported in rodents, where IAT is the adipose tissue that is most susceptible to browning (Petrov et al., 2015). However, results showing induction of browning in comparison to subcutaneous fat go in the same direction as data obtained in humans, indicating that adipocytes surrounding the aorta present markers of major thermogenic activity than adipocytes coming from subcutaneous adipose tissue (Vargas et al., 2018). Thus, our current data reinforce the use of ferret as an interesting animal model to mimic human browning in comparison to rodents.

In summary, the most relevant result from our study is the higher browning capacity of tPVAT in response to cold exposure in ferrets, which is accompanied by a profound immunosuppression in this depot. Taking into consideration the known protective cardiovascular effect of both aspects this animal model appears as very promising to perform cardiovascular disease research more likely to be extrapolated to humans than research performed with rodents.

## Ethics Statement

This study was carried out in accordance with the recommendations of Directive 2010/63/EU of the European Parliament on the protection of animals used for scientific purposes. The protocol was approved by the Bioethical Committee of the University of the Balearic Islands.

## Author Contributions

AP and PO conceived and designed the experiments. BR, EC, and PO carried out experimental procedures. BR, ES, JK, EC, PO, and AP participated in the data analysis and interpretation. BR and PO wrote the manuscript. All authors revised the definitive version, read and approved the final manuscript.

## Conflict of Interest Statement

The authors declare that the research was conducted in the absence of any commercial or financial relationships that could be construed as a potential conflict of interest.
