# Supplementary material for: Cold Induced Depot-Specific Browning in Ferret Aortic Perivascular Adipose Tissue
Source: Front Physiol. 2019 Sep 18;10:1171. doi: 10.3389/fphys.2019.01171 (PMC6759601; doi:10.3389/fphys.2019.01171)
Supplement: Supplementary file 1 [file Data_Sheet_1.PDF]

Fifty top up-regulated genes at 4℃ in the tPVAT.

Sequence ID	Gene symbol	Gene name	p-value	Fold change
Carbohydrate metabolism				
XM_004773546.1	*Galnt15*	*Polypeptide N-Acetylgalactosaminyltransferase 15*	5.38E-03	4.86
XM_004741919.1	*Cav1*	*Caveolin 1*	1.07E-03	3.44
Cell cycle				
XM_004741923.1	*Cav2*	*Caveolin 2*	1.33E-03	3.42
XM_004780198.1	*Cebpd*	*Ccaat/Enhancer Binding Protein (C/Ebp), Delta*	2.50E-03	2.76
XM_004740346.1	*Ctgf*	*Connective Tisuue Growth Factor*	3.87E-03	3.08
XM_004768767.1	*Errfi1*	*Erbb Receptor Feedback Inhibitor 1*	4.51E-03	3.50
XM_004737947.1	*Ghr*	*Growth Hormone Receptor*	5.30E-03	3.09
XM_004758546.1	*Ndrg2*	*Ndrg Family Member 2*	7.65E-03	2.91
XM_004765797.1	*Adirf*	*Adipogenesis Regulatory Factor*	2.76E-02	2.68
Cell differentiation and development				
XM_004755807.1	*Gprc5a*	*G Protein-Coupled Receptor, Class C, Group 5, Member A*	9.84E-03	3.56
XM_004782340.1	*Loc101676727*	*Cytochrome P450 3a12-Like*	3.02E-03	3.74
Cell protection				
XM_004744215.1	*Loc101680117*	*Metallothionein-1e-Like*	2.51E-03	4.61
XM_004766404.1	*Loc101687859*	*Selenoprotein M*	6.16E-03	2.76
XM_004757964.1	*Loc101692625*	*Metallothionein-1e Pseudogene*	2.17E-03	5.33
XM_004762982.1	*Gadd45a*	*Growth Arrest And Dna Damage Inducible Alpha*	3.79E-03	2.74
Cell stress response				
XM_004740244.1	*Sgk1*	*Serum/Glucocorticoid Regulated Kinase 1*	1.34E-05	4.85
XM_004774974.1	*Cyth3*	*Cytohesin 3*	7.87E-05	2.93
Cell trafficking				
XM_004764475.1	*Fcho2*	Fch Domain Only 2	9.42E-04	2.63
XM_004743651.1	*Mal2*	*Mal, T-Cell Differentiation Protein 2*	4.57E-02	3.71
XM_004766342.1	*Scarb2*	*Scavenger Receptor Class B, Member 2*	2.12E-03	2.84
XM_004753054.1	*Il1r2*	*Interleukin 1 Receptor, Type Ii*	3.45E-05	4.29
Immune response				
XM_004771986.1	*Nfkbiz*	*Nuclear Factor Of Kappa Light Polypeptide Gene Enhancer In B Cells Inhibitor, Zeta*	7.23E-03	2.84
XM_004739946.1	*Trem2*	*Triggering Receptor Expressed On Myeloid Cells 2*	1.58E-02	2.73
XM_004747736.1	*Abca1*	*Atp-Binding Cassette, Subfamily A (Abc1), Member 1*	7.23E-03	3.33
Lipid metabolism				
XM_004743410.1	*Dgkb*	*Diacylglycerol Kinase, Beta*	1.56E-02	2.80
XM_004774219.1	*Fabp4*	*Fatty Acid Binding Protein 4, Adipocyte*	1.38E-02	2.95
XM_004771705.1	*Retn*	*Resistin*	2.84E-03	2.72
XM_004766965.1	*Atp6v0d2*	*Atpase, H+ Transporting, Lysosomal V0 Subunit D2*	1.02E-02	2.72
Others				
XM_004780129.1	*Amy2a*	*Amylase, alpha 2A*	1.24E-03	2.92
XM_004776777.1	*Ctsk*	*Cathepsin K*	9.26E-03	3.92
XM_004755799.1	*Emp1*	*Epithelial Membrane Protein 1*	3.47E-02	3.52
XM_004766844.1	*F2rl2*	*Coagulation Factor Ii (Thrombin) Receptor-Like 2*	2.39E-03	4.06
XM_004752563.1	*F3*	*Coagulation Factor Iii (Thromboplastin, Tissue Factor)*	6.46E-05	4.06
XM_004755885.1	*Fibin*	*Fin Bud Initiation Factor Homolog (Zebrafish)*	4.13E-04	3.26
XM_004775062.1	*Klf5*	*Kruppel-Like Factor 5 (Intestinal)*	2.73E-02	3.41
XM_004769265.1	*Pam*	*Peptidylglycine Alpha-Amidating Monooxygenase*	8.35E-04	2.82
XM_004767845.1	*Pdk4*	*Pyruvate Dehydrogenase Kinase, Isozyme 4*	6.41E-03	5.73
XM_004759991.1	*Rbbp8nl*	*Rbbp8 N-Terminal Like*	1.88E-03	3.56
XM_004737647.1	*Wwc1*	*Ww And C2 Domain Containing 1*	2.85E-03	2.98
XM_004760169.1	*Dhrs7c*	Dehydrogenase/Reductase (Sdr Family) Member 7c	4.41E-02	2.58
Redox metabolism				
XM_004756692.1	*Fmo2*	*Flavin Containing Monooxygenase 2*	7.37E-03	2.94
XM_004761383.1	*Upk1b*	Uroplakin 1b	5.50E-03	2.57
Signal transduction				
XM_004741918.1	*Met*	*Met Proto-Oncogen*	2.17E-03	3.05
XM_004772400.1	*Dst*	*Dystonin*	7.30E-04	3.60
Structure/cytoskeleton				
XM_004742842.1	*Lama3*	*Laminin, Alpha 3*	5.56E-03	3.14
XM_004761362.1	*Lox*	*Lysyl Oxidase*	3.94E-03	2.75
XM_004766755.1	*Myom2*	*Myomesin 2*	2.71E-02	2.76
XM_004756105.1	*Spp1*	*Secreted Phosphoprotein 1*	7.16E-03	3.39
Unknown				
XM_004777698.1	*Loc101672612*	Chromosome Unknown Open Reading Frame, Human C8orf4	1.46E-03	2.63
XM_004764364.1	*Loc101686598*	*Chromosome Unknown Open Reading Frame, Human C2orf40*	1.90E-02	3.09

Fity top down-regulated genes at 4ºC in the tPVAT.

Sequence ID	Gene symbol	Gene name	p-value	Fold change
Cell cycle				
XM_004737693.1	*Ccnjl*	*Cyclin J-Like*	8.35E-04	-7.68
XM_004766651.1	*Cdca3*	*Cell Division Cycle Associated 3*	1.22E-03	-4.61
XM_004740751.1	*Cdca8*	*Cell Division Cycle Associated 8*	3.79E-04	-4.44
XM_004774496.1	*Cdkn2c*	*Cyclin-Dependent Kinase Inhibitor 2c (P18, Inhibits Cdk4)*	2.05E-04	-5.05
XM_004741203.1	*E2f2*	*E2f Transcription Factor 2*	3.77E-05	-4.40
XM_004740483.1	*Kif2c*	*Kinesis Family Member 2 C*	4.35E-04	-5.06
XM_004751880.1	*Mki67*	*Antigen Identified By Monoclonal Antibody Ki-67*	4.88E-04	-6.53
XM_004745791.1	*Rrm2*	*Ribonucleotide Reductase M2*	9.91E-04	-4.66
XM_004748404.1	*Spc24*	*Spc24, Ndc80 Kinetochore Complex Component*	1.07E-03	-4.82
XM_004741128.1	*Stmn1*	*Stathmin1*	3.14E-03	-5.62
XM_004742802.1	*Tyms*	*Thymidylate Synthetase*	3.77E-04	-4.62
XM_004771486.1	*Uhrf1*	*Ubiquitin-Like With Phd And Ring Finger Domain 1*	5.42E-03	-6.10
Immune response				
XM_004771683.1	*Ccl25*	*Chemokine (C-C Motif) Ligand 25*	3.29E-03	-6.86
XM_004760815.1	*Ccr9*	*Chemokine (C-C Motif) Receptor 9*	7.86E-03	-7.07
XM_004775803.1	*Cd1d*	*Cd1d Molecule*	8.33E-03	-7.81
XM_004775804.1	*Cd1e*	*Cd1e Molecule*	5.26E-03	-5.60
XM_004749949.1	*Cd3g*	*Cd3g Molecule, Gamma (Cd3-Tcr Complex)*	2.12E-02	-4.94
XM_004741105.1	*Cd52*	*Cd52 Molecule*	2.69E-03	-6.13
XM_004742367.1	*Cd8a*	*Cd8a Molecule*	1.87E-02	-5.42
XM_004762280.1	*Itgb2*	*Integrin, Beta 2 (Complement Component 3 Receptor 3 And 4 Subunit)*	1.50E-02	-4.57
XM_004740918.1	*Lck*	*Lymphocyte-Specific Protein Tyrosine Kinase*	7.00E-03	-5.35
XM_004775799.1	*Loc101690640*	*T-Cell Surface Glycoprotein Cd1a-Like*	3.52E-03	-7.85
XM_004778740.1	*Prss16*	*Protease, Serine 16 (Thymus)*	9.38E-03	-9.75
XM_004739882.1	*Ptcra*	*Pre T-Cell Antigen Receptor Alpha*	2.72E-03	-7.42
XM_004756002.1	*Rag1*	*Recombination Activating Gene 1*	1.26E-02	-7.63
XM_004756007.1	*Rag2*	*Recombination Activating Gene 2*	8.93E-03	-5.74
XM_004769506.1	*Tbata*	*Thymus, Brain And Testes Associated*	5.53E-04	-9.81
XM_004740174.1	*Themis*	*Thymocyte Selection Associated*	8.36E-03	-4.93
Lipid metabolism				
XM_004773512.1	*Dgkd*	*Diacylglycerol Kinase, Delta*	1.82E-03	-4.48
XM_004748113.1	*Loc101691931*	*Cytochrome P450 2u1-Like*	7.29E-03	-5.76
Others				
XM_004741734.1	*Akr1b1*	*Ldo-Keto Reductase Family 1, Member B1 (Aldose Reductase)*	3.83E-03	-6.46
XM_004741682.1	*Atp6v0a4*	*Atpare, H+ Transporting, Lysosomal V0 Subunit A4*	5.70E-04	-5.41
XM_004768567.1	*Cdh17*	*Cadherin 17*	2.00E-03	-5.79
XM_004738703.1	*Chchd6*	*Coiled-Coil-Helix-Coiled-Coil-Helix Domain*	2.22E-03	-4.87
XM_004749590.1	*Dntt*	*Dna Nucleotidylexotransferase*	1.29E-02	-5.76
XM_004770551.1	*Drd5*	*Dopamine Receptor D5*	2.76E-03	-5.00
XM_004774882.1	*Loc101676398*	*Phytanoyl-Coa Dioxygenase, Peroxisomal -Like*	1.28E-03	-6.53
XM_004778790.1	*Loc101683777*	*Histone H4 Type Viii-Like*	8.09E-03	-5.12
XM_004781521.1	*Loc101690749*	*Histone H4-Like*	6.50E-03	-5.20
XM_004782596.1	*Loc101690963*	*Phytanoyl-Coa Dioxygenase, Peroxisomal-Like*	1.54E-03	-6.01
XM_004774884.1	*Phyh*	*Phytanoyl-Coa 2-Hydroxylase*	5.60E-04	-5.88
XM_004743585.1	*Sla*	*Src-Like-Adaptor*	1.82E-02	-5.29
Protein metabolism/degradation				
XM_004754074.1	*Ctrb1*	*Chymotrypsinogen B1*	6.23E-03	-6.11
XM_004750821.1	*Loc101674311*	*Putative Serine Protease 29-Like*	1.24E-03	-7.01
XM_004742552.1	*Serpind1*	*Serpin Peptidase Inhibitor, Clade D (Heparin Cofactor), Member 1*	3.86E-04	-4.63
XM_004746330.1	*Ube2c*	*Ubiquitin-Conjudatng Enzyme E2c*	4.78E-04	-4.87
Signal transduction				
XM_004743172.1	*Rac2*	*Ras-Related C3 Botulinum Substrate 2*	2.24E-02	-4.50
XM_004767912.1	*Rgs16*	*Regulator Of G-Protein Signaling 16*	3.44E-03	-4.69
Unknown				
XM_004778789.1	*Loc101683478*	*Uncharacterized*	9.84E-03	-9.84
XM_004754742.1	*Loc101694602*	*Uncharacterized*	4.52E-04	-5.57

Fifty top up-regulated genes at 4ºC in the IAT.

Sequence ID	Gene symbol	Gene name	p-value	Fold change
Amino acid metabolism				
XM_004757114.1	*Ass1*	*Argininosuccinate Synthase 1*	6.19E-03	4.53
XM_004763011.1	*Cth*	*Cystathionase (Cystathionine Gamma-Lyase)*	1.79E-02	3.00
XM_004767902.1	*Glul*	*Glutamate-Ammonia Ligase*	2.32E-02	2.43
Calcium transport				
XM_004763794.1	*Alpk3*	*Alpha-Kinase 3*	3.91E-02	3.20
XM_004773978.1	*Atp2a1*	*Atpase, Ca++ Transporting, Cardiac Muscle, Fast Twitch 1*	4.40E-02	2.83
XM_004756257.1	*Cacna1s*	*Calcium Channel, Voltage-Dependent, L Type, Alpha 1S Subunit*	4.67E-02	4.64
XM_004743708.1	*Ramp1*	*Receptor (G Protein-Coupled) Activity Modifying Protein 1*	3.99E-02	2.49
XM_004740339.1	*Trdn*	*Triadin*	2.81E-02	3.18
Cell protection				
XM_004769485.1	*Ddit4*	*DNA-Damage-Inducible Transcript 4*	2.12E-02	2.59
XM_004778803.1	*Gadd45g*	*Growth Arrest And DNA-Damage-Inducible, Gamma*	2.68E-02	3.47
XM_004757923.1	*Net1*	*Neuroepithelial Cell Transforming 1*	5.40E-03	3.36
XM_004749869.1	*Nnmt*	*Nicotinamide N-Methyltransferase*	4.74E-03	2.44
Electron transport chain in mitochondria/oxidative phosphorylation				
XM_004762520.1	*Loc101690488*	*Cytochrome C-Like*	2.43E-02	2.68
XM_004756805.1	*Loc101686662*	*Cytochrome C Oxidase Protein 20 Homolog*	3.94E-02	2.39
XM_004781049.1	*Loc101674825*	*Cytochrome C Oxidase Subunit 8B, Mitochondrial-Like*	4.42E-02	3.22
XM_004757734.1	*Slc25a4*	*Solute Carrier Family 25, Member 4*	2.29E-02	2.44
FA transport				
XM_004740951.1	*Fabp3*	*Fatty Acid Binding Protein 3, Muscle And Heart*	1.57E-02	3.10
XM_004767850.1	*Pon3*	*Paraoxonase 3*	1.44E-04	6.74
Gene expression regulation				
XM_004780198.1	*Cebpd*	*CCAAT/Enhancer Binding Protein (C/EBP), Delta*	3.91E-02	2.43
XM_004740700.1	*Heyl*	*Hairy/Enhancer-Of-Split Related With YRPW Motif-Like*	3.21E-02	2.38
XM_004743220.1	*Maff*	*V-Maf Avian Musculoaponeurotic Fibrosarcoma Oncogene Homolog F*	4.69E-02	2.36
XM_004781266.1	*Myf6*	*Myogenic Factor 6*	2.26E-02	2.72
Ion transport				
XM_004762066.1	*Atp1b4*	*Atpase, (Na+)/K+ Transporting, Beta 4 Polypeptide*	3.99E-02	3.75
XM_004750299.1	*Tmc5*	*Transmembrane Channel-Like 5*	4.22E-04	3.59
Others				
XM_004751192.1	*Capn3*	*Calpain 3*	2.91E-02	2.96
XM_004759150.1	*Ckmt2*	*Creatine Kinase, Mitochondrial 2, Sarcomeric*	1.97E-02	4.07
XM_004770688.1	*Fkbp5*	*FK506 Binding Protein 5*	6.83E-03	3.10
XM_004752563.1	*F3*	*Coagulation Factor III (Thromboplastin, Tissue Factor)*	1.51E-02	3.32
XM_004741401.1	*Hspb7*	*Heat Shock Protein Family, Member 7 (Cardiovascular)*	2.52E-02	7.24
XM_004738170.1	*Itih4*	*Inter-Alpha-Trypsin Inhibitor Heavy Chain Family Member 4*	2.15E-02	2.34
XM_004743718.1	*Klhl30*	*Kelch-Like Family Member 30*	2.98E-02	4.66
XM_004770231.1	*Loc101676773*	*HRAS-Like Suppressor 3*	2.06E-02	2.34
XM_004757964.1	*Loc101692625*	*Metallothionein-1-Like*	4.64E-02	5.45
XM_004755129.1	*Loc101674041*	*Putative G Antigen Family E Member 3-Like*	4.23E-02	2.36
XM_004741999.1	*Loc101681100*	*Transmembrane Protein 229A-Like*	8.19E-03	2.37
XM_004755772.1	*Pde6h*	*Phosphodiesterase 6H, Cgmp-Specific, Cone, Gamma (Pde6h), Mrna*	2.90E-02	2.82
XM_004777369.1	*Rasgef1b*	*Rasgef Domain Family, Member 1B*	1.56E-02	2.65
XM_004750746.1	*Rpl3l*	*Ribosomal Protein L3-Like*	1.18E-02	2.68
XM_004769189.1	*Smpx*	*Small Muscle Protein, X-Linked*	4.32E-02	6.00
XM_004768064.1	*Ucp3*	*Uncoupling Protein 3 (Mitochondrial, Proton Carrier)*	1.03E-02	3.06
Signal transduction				
XM_004775565.1	*Asb5*	*Ankyrin Repeat And SOCS Box-Containing 5*	3.08E-02	3.72
XM_004775460.1	*Mapk12*	*Mitogen-Activated Protein Kinase 12*	2.03E-02	2.43
XM_004756052.1	*Pdlim5*	*PDZ And LIM Domain 5*	1.77E-02	3.50
XM_004740244.1	*Sgk1*	*Serum/Glucocorticoid Regulated Kinase 1*	3.66E-02	2.65
XM_004746413.1	*Sgk2*	*Serum/Glucocorticoid Regulated Kinase 2*	1.43E-02	2.74
Structure/cytoskeleton				
XM_004774791.1	*Actn2*	*Actinin Alpha 2*	3.69E-02	7.62
XM_004741862.1	*Lmod2*	*Leiomodin 2 (Cardiac)*	3.68E-02	7.05
XM_004747997.1	*Myoz2*	*Myozenin 2*	1.07E-02	4.93
XM_004776741.1	*Tmod4*	*Tropomodulin 4*	4.88E-02	3.33
XM_004744005.1	*Xirp2*	*Xin Actin-Binding Repeat Containing 2*	1.58E-02	5.56

Fifty top down-regulated genes at 4°C in the IAT.

Sequence ID	Gene symbol	Gene name	p-value	Fold change
Cell cycle				
XM_004772789.1	*Fstl1*	*Follistatin-Like 1*	1.73E-03	-2.22
XM_004742992.1	*Igf1*	*Insulin-Like Growth Factor 1*	4.76E-03	-2.97
Cell differentiation and development				
XM_004772230.1	*Cpz*	*Carboxypeptidase Z*	5.52E-04	-4.32
XM_004739323.1	*Dact1*	*Dishevelled-Binding Antagonist Of Beta-Catenin 1*	8.43E-04	-2.36
XM_004746553.1	*Gdf5*	*Growth Differentiation Factor 5*	5.21E-03	-2.97
XM_004749459.1	*Kazald1*	*Kazal-Type Serine Peptidase Inhibitor Domain 1*	8.24E-03	-2.22
XM_004754862.1	*Loc101677386*	*Protein Delta Homolog 1-Like*	9.74E-03	-2.52
XM_004761315.1	*Nrep*	*Neuronal Regeneration Related Protein*	5.91E-04	-2.25
XM_004772002.1	*Sfrp2*	*Secreted Frizzled-Related Protein 2*	1.03E-02	-3.97
XM_004739496.1	*Sfrp4*	*Secreted Frizzled-Related Protein 4*	4.49E-02	-2.53
XM_004740360.1	*Trabd2b*	*Trab Domain Containing 2B*	6.23E-03	-2.38
XM_004741795.1	*Kcp*	*Kielin/Chordin-Like Protein*	7.26E-04	-2.60
XM_004739586.1	*Lrrc17*	*Leucine Rich Repeat Containing 17*	2.80E-03	-2.80
Food intake control				
XM_004754240.1	*Cck*	*Cholecystokinin*	6.93E-03	-4.64
XM_004741831.1	*Lep*	*Leptin*	1.84E-02	-4.28
Others				
XM_004757188.1	*Cercam*	*Cerebral Endothelial Cell Adhesion Molecule*	1.53E-03	-2.39
XM_004763149.1	*Creb3l1*	*Camp Responsive Element Binding Protein 3-Like 1*	4.27E-03	-2.88
XM_004741584.1	*Loc101682426*	*Family with sequence similarity 43, member B*	1.46E-02	-2.23
XM_004765489.1	*Qpct*	*Glutaminyl-Peptide Cyclotransferase*	1.46E-03	-2.35
XM_004769061.1	*Rgag1*	*Retrotransposon Gag Domain Containing 1*	3.31E-02	-3.04
XM_004754890.1	*Srpx*	*Srpxsushi-Repeat-Containing Protein, X-Linked*	1.31E-03	-2.27
XM_004779817.1	*Loc101684445*	*Leukocyte Immunoglobulin-Like Receptor Subfamily A Member 6*	1.70E-02	-2.22
Signal transduction				
XM_004743168.1	*C1qtnf6*	*C1q And Tumor Necrosis Factor Related Protein 6*	3.53E-03	-2.33
XM_004741223.1	*Ephb2*	*Eph Receptor B2*	6.31E-03	-2.22
XM_004771853.1	*Klhl31*	*Kelch-Like Family Member 31*	1.17E-02	-2.75
XM_004739125.1	*Tshr*	*Thyroid Stimulating Hormone Receptor*	2.11E-02	-3.75
Structure/cytoskeleton				
XM_004758676.1	*Cilp*	*Cartilage Intermediate Layer Protein, Nucleotide Pyrophosphohydrolase*	1.89E-03	-3.13
XM_004743495.1	*Col14a1*	*Collagen, Type XIV, Alpha 1*	4.70E-04	-2.99
XM_004740947.1	*Col16a1*	*Collagen, Type XVI, Alpha 1*	4.48E-03	-2.46
XM_004763363.1	*Col3a1*	*Collagen, Type III, Alpha 1*	1.93E-04	-8.58
XM_004743676.1	*Col6a3*	*Collagen, Type VI, Alpha 3*	6.77E-03	-2.31
XM_004759996.1	*Col9a3*	*Collagen, Type IX, Alpha 3*	1.99E-03	-2.26
XM_004768864.1	*Cthrc1*	*Collagen Triple Helix Repeat Containing 1*	3.34E-03	-3.55
XM_004748554.1	*Dcn*	*Decorin*	4.49E-03	-2.53
XM_004772734.1	*Fkbp10*	*FK506 Binding Protein 10*	2.71E-03	-2.40
XM_004756352.1	*Fmod*	*Fibromodulin*	6.11E-03	-2.76
XM_004766993.1	*Fn1*	*Fibronectin 1*	1.78E-03	-3.62
XM_004764820.1	*Loc101686788*	*Collagen Alpha-1(I) Chain-Like*	6.74E-03	-4.41
XM_004767862.1	*Loc101690329*	*Collagen Alpha-2(I) Chain-Like*	7.40E-04	-6.23
XM_004763267.1	*Loxl2*	*Lysyl Oxidase-Like 2*	1.03E-03	-2.52
XM_004748555.1	*Lum*	*Lumican*	1.27E-04	-6.00
XM_004752504.1	*Mfap5*	*Microfibrillar Associated Protein 5*	2.84E-02	-2.26
XM_004781342.1	*Mmp23b*	*Matrix Metallopeptidase 23B*	1.60E-03	-2.36
XM_004775945.1	*Mpz*	*Myelin Protein Zero*	9.73E-04	-4.06
XM_004749294.1	*Mrc2*	*Mannose Receptor, C Type 2*	1.57E-03	-2.68
XM_004761526.1	*Pcolce*	*Procollagen C-Endopeptidase Enhancer*	1.74E-03	-3.24
XM_004779534.1	*Postn*	*Periostin, Osteoblast Specific Factor*	6.20E-04	-4.88
XM_004768014.1	*Serpinh1*	*Serpin Peptidase Inhibitor, Clade H (Heat Shock Protein 47), Member 1, (Collagen Binding Protein 1)*	2.64E-03	-2.68
XM_004778593.1	*Thbs3*	*Thrombospondin 3*	4.35E-04	-2.64
Unknown				
XM_004753470.1	*Loc101686176*	*Uncharacterized LOC101686176*	1.18E-02	-2.35
